# Overview of Cotinine Cutoff Values for Smoking Status Classification

**DOI:** 10.3390/ijerph13121236

**Published:** 2016-12-14

**Authors:** Sungroul Kim

**Affiliations:** Department of Environmental Health Sciences, Soonchunhyang University, Asan 31538, Korea; sungroul.kim@gmail.com; Tel.: +82-41-530-1249

**Keywords:** cutoff value, cotinine, smoking status, biomarker

## Abstract

While cotinine is commonly used as a biomarker to validate self-reported smoking status, the selection of an optimal cotinine cutoff value for distinguishing true smokers from true nonsmokers shows a lack of standardization among studies. This review describes how the cutoff values have been derived, and explains the issues involved in the generalization of a cutoff value. In this study, we conducted an English-language literature search in PubMed using the keywords “cotinine” and “cutoff” or “self-reported” and “smoking status” and “validation” for the years 1985–2014. We obtained 104 articles, 32 of which provided (1) sensitivity and specificity of a cutoff value and (2) determination methods for the given cutoff value. We found that the saliva cotinine cutoff value range of 10–25 ng/mL, serum and urine cotinine cutoff of 10–20 ng/mL and 50–200 ng/mL, respectively, have been commonly used to validate self-reported smoking status using a 2 × 2 table or a receiver operating characteristics (ROC) curve. We also found that recent large population-based studies in the U.S. and UK reported lower cutoff values for cotinine in serum (3 ng/mL) and saliva (12 ng/mL), compared to the traditionally accepted ones (15 and 14 ng/mg, respectively).

## 1. Introduction

Accurate assessment of smoking status is critical for documenting the extent of the tobacco epidemic, estimating population risk and smoking-attributable disease burden, and evaluating the progress of tobacco control programs throughout the world [1,2,3,4]. A standardized questionnaire has been the most commonly used tool to assess smoking prevalence and exposure to passive smoke.

Self-reported smoking status, however, may not always represent a subject’s true smoking status; limitations in recall may affect details of past smoking and respondent bias may lead to inaccurate reporting of current or past use. The extent of respondent bias may increase over time as changing social norms make tobacco use less acceptable. Furthermore, a questionnaire may not be completely accurate for evaluating the passive smoke exposure of nonsmokers. Additionally, determining the smoking status of recent former smokers, whose exposure levels to passive smoke vary, is even more challenging when questionnaires are the only available tools [5,6].

For these reasons, biological markers of tobacco exposure have been used in prevalence surveys and epidemiologic studies for many years to validate reported smoking status [2,7,8,9,10,11,12,13], and to monitor and track population exposure to tobacco with respect to people, place, and time. Biomarkers may include the concentrations of smoke-related components, their metabolites, or the products of interaction between chemicals in smoke and target molecules in biological materials [13].

Nicotine is a tobacco-specific biomarker, as are its metabolites [14,15,16]. Once the nicotine is absorbed as bound to particles or in vapor phase [17,18], it is primarily metabolized to cotinine in the liver [14,15]; its half-life in the body is approximately 2–3 h, while that of cotinine is 12–20 h [19,20,21]. A small amount of cotinine (10%–15%) is excreted in urine, and the remaining cotinine is further metabolized to trans-3’-hydroxycotinine and other byproducts [14]. Because of its longer half-life, cotinine, measured in saliva, urine, or serum, is a commonly used biomarker of tobacco smoke exposure. A comprehensive review on biomarkers of tobacco smoke exposure was published in 1999 [8]. Levels of these biomarkers are affected by processes of uptake, distribution, metabolism, and elimination of the chemicals in the body after exposure has occurred.

In a population that includes smokers and nonsmokers, the distribution of a biomarker specific to tobacco smoke, e.g., nicotine or cotinine, would be expected to be bimodal, and reflective of two underlying distributions: for nonsmokers and smokers. In a general dichotomous template for the comparison of self-reported smoking status with a biomarker, we can calculate two key self-report validity indicators: sensitivity (%), the percentage of self-reported smokers classified as smokers by biomonitoring; and specificity (%), the percentage of self-reported nonsmokers classified as nonsmokers by biomonitoring. Of particular concern is the rate of under-reporting or “false-negatives”: those who report as nonsmokers but have a positive biomarker level. In general, nonsmokers are not expected to report themselves as smokers, but because of exposure to passive smoke, it is possible to have a positive biomarker level, which makes the specificity less than 100%. The lower the specificity for exposure status is, the greater the underestimation of the association between exposure and disease occurrence [22].

As mentioned above, the distribution of the marker in nonsmokers would reflect the pattern of passive smoking, while the distribution in active smokers would reflect their tobacco use. In setting a cutoff value, the goal is to optimize sensitivity and specificity, although inevitably there is a tradeoff between specificity and sensitivity. In a bimodal distribution, generally, a lower cutoff value achieves higher sensitivity, but has lower specificity than a higher value, i.e., a higher number of nonsmokers exhibit biomarker levels above the cutoff. A higher cutoff value has greater specificity but lower sensitivity than a lower cutoff value.

Ideally, the selection of a cutoff value would take into consideration the underlying distributions of the marker in nonsmokers and smokers. However, the distribution may change over time; with effective tobacco control, the distribution of values in nonsmokers would shift to the low side, and more nonsmokers would have a non-detectable level of the marker. With such a shift, the cutoff point may also be shifted to the low value. The distributions are also likely to vary from population to population because of differing patterns of active smoking and passive smoke exposure.

Cotinine is widely used as a validation standard for classifying exposure status [12,23,24,25,26,27,28,29,30,31,32]. Furthermore, a subcommittee of the Society for Research on Nicotine and Tobacco (SRNT) evaluated the utility of cotinine, carbon monoxide (CO), and thiocyanate (SCN) as biomarkers of tobacco use and cessation, and recommended for their application in clinical trials with cutoff values [33].

However, previous studies have showed a lack of standardization among studies in the selection of an optimal cotinine cutoff value for distinguishing true smokers from true nonsmokers. In addition, limited studies assessed whether cutoff values have changed over time. This review describes how cutoff values have been derived, assesses the changes in cutoff values using results obtained from large population-based studies, and explains the issues in the cutoff value determination process.

## 2. Materials and Methods

For this summary, articles in English using the keywords “cotinine” and “cutoff” or “self-reported” and “smoking status” and “validation” were identified for review in the public literature database, PubMed, for the years 1985 to 2014 (N = 104). The articles were eligible for inclusion if (1) the results provided information on agreement and discrepancy between self-reported smoking status and cotinine test using self-reporting or cotinine test as the gold standard; and (2) the results described determination methods for a given cutoff value of cotinine concentration. The bibliographies of texts were also searched to identify cited methods for cutoff determination. We included results from adolescent study populations to compare with those of adults. We excluded articles describing a cotinine test strip or a cotinine dipstick method. In total, 32 articles were included in this review.

We summarized cutoff values, their determination methods, and study population characteristics. Then, we summarized the sensitivity and specificity for discriminating smokers and nonsmokers with self-reporting as a gold standard test using three biospecimens: saliva, serum, and urine. In addition, we evaluated the change in cutoff values over time and their differences with respect to populations and regions. Furthermore, we describe issues in the cutoff value determination process for generalization purposes.

## 3. Results

Study population characteristics, methods used for determining cutoffs, the cotinine cutoff values in saliva, serum, and urine, and the number of self-reported smokers and nonsmokers are summarized in Table 1, Table 2 and Table 3. Most studies were conducted in countries in North America and Europe between mid-1980 and 2013, with sample sizes ranging from 69 to 24,332.

The cutoff values were selected using a variety of methods, including the maximization of sensitivity and specificity using cotinine levels in nonsmokers [7], passive smokers [34], or smokers [35]. They were also determined using a 2 × 2 table [27,28,30,31,36]. More recently, the cutoff value has been obtained using a receiver operating characteristics (ROC) curve, an approach to simultaneously optimize sensitivity and specificity and provide the highest percentage of correctly classified smoking status [23,25,37,38,39].

### 3.1. Salivary Cotinine

**Figure 1:**
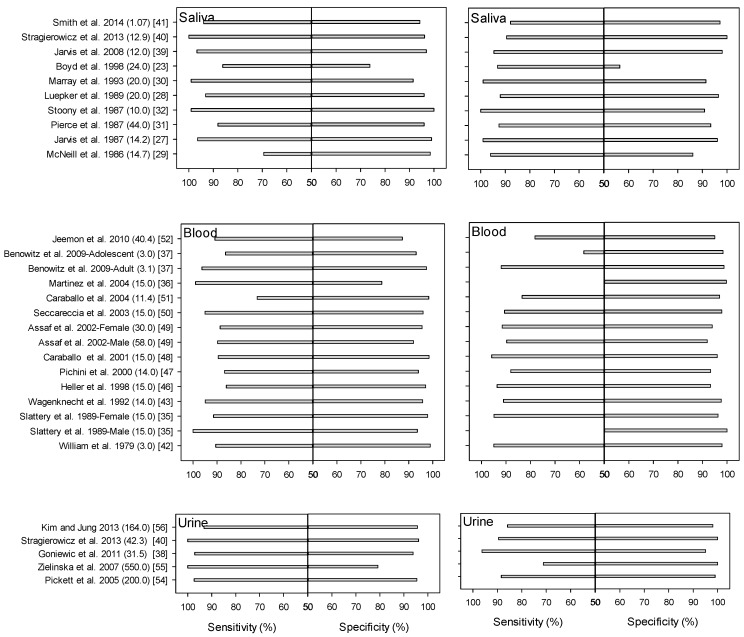
Plots of sensitivities and specificities according to the cutoff values of salivary or serum cotinine. (The numbers in parentheses represent cutoff values and citation number). Gold Standard: Self-Report (Left); Cotinine (Right).

Among the 11 studies identified, cutoff values of salivary cotinine for differentiating smokers from non-smokers ranged from 10 to 44 ng/mL. The overall ranges of sensitivity and specificity were 69% to 99% and 74% to 99%, respectively (Figure 1). Jarvis et al. (1987) studied 211 adult outpatients in London, UK, and reported a salivary cotinine cutoff value of 14.2 ng/mL [27]. This value was selected using a 2 × 2 table (Table 1). The sensitivity and specificity of the value were 96.4% and 99.0%, respectively. Pierce et al. (1987) reported 44 ng/mL as the cutoff value in an Australian study population (N = 975) aged 14 years or older [31]. Etzel (1990) introduced a cutoff value of 10 ng/mL after reviewing the results of 22 studies selected from an English-language MEDLINE search for the years 1973–1989 using “saliva” and “cotinine” as keywords [12]. The cutoff value of 10 ng/mL was determined by comparing the distribution of salivary cotinine with respect to smoking status: values for passive smokers were below 5 ng/mL; heavy passive smokers exhibited a value of 10 ng/mL or slightly higher; infrequent regular smokers were between 10 and 100 ng/mL; and regular active smokers exhibited levels of 100 ng/mL or higher. However, the sensitivity and specificity for the 10 ng/mL value determined by Etzel (1990) were not available. Recently, Jarvis et al. (2008) [39] reported a new salivary cotinine cutoff value of 12 ng/mL using an ROC curve, with a sensitivity of 96.7% and specificity of 96.9% for distinguishing cigarette smokers from never smokers among participants aged 16 years or older in the Health Survey for England (HSE) for the years 1996–2004. Figure 1 shows the distributions of sensitivity and specificity values for the various cotinine cutoff levels summarized in Table 1, Table 2 and Table 3.

### 3.2. Serum Cotinine

Fourteen studies were identified for evaluation of serum cotinine cutoff values. The cutoff values ranged from 3.0 to 20 ng/mL, yielding a sensitivity range of 73.2% to 98.9% and a specificity range of 78.7% to 99.0%. Eight out of the fourteen studies provided a cutoff value of 14 or 15 mg/mL between 1989 and 2004 (Table 2). Slattery et al. (1989) [35] identified a cutoff value of 15 ng/mL, which was equal to 6% of the mean serum cotinine level of smokers in their study. Pirkle et al. (1996) [44] selected 15 ng/mL as a serum cotinine cutoff value as it represented the separation point in the bimodal distribution of serum cotinine levels in tobacco users and nonusers who participated in the Third National Health and Nutrition Examination Survey (NHANES III, 1988–1991) in the U.S. (N = 10,270, aged 4 years or older). Information on sensitivity and specificity was not available for Pirkle’s study. The value of 15 ng/mL obtained from the NHANES study, based on a relatively large number of healthy participants with diverse racial makeup and wide age range, was consistent with the cutoff value from an ROC curve of the Italian population that participated in a cross-sectional study on cardiovascular disease (MATISS project, N = 3379) [50]. Using the NHANES III dataset but with an extended survey period (1988–1994) and adult participants aged 17 years or older (N = 15,357), Caraballo et al. (2001) [48] validated and provided a new sensitivity (89.5%) and specificity (98.5%) using the cutoff value determined by Pirkle et al. (1996) [44]. A few years later, Caraballo et al. [51] reported their own new cutoff (11.4 ng/mL) with corresponding sensitivity (73.2%) and specificity (98.4%) for the adolescent population (N = 2107) in the same dataset (NHANES III, 1988–1994) using an ROC curve. With few exceptions [42,49,52], the serum cotinine cutoff values in Table 3 were comparable, and fell within a range of 8 to 20 ng/mL. However, Benowitz et al. [37] recently reported much smaller cutoff values of 3.08 and 2.99 ng/mL for adults and adolescents, respectively, by ROC analysis of data from NHANES for 1999–2004.

### 3.3. Urinary Cotinine

Only a few studies (N = 5) were included in our review, and cutoff values ranged between 31.5 and 550 ng/mL [34,38,53,54,55]. Pickett et al. (2005) provided a cutoff value of 200 ng/mL using an ROC curve on data from 998 pregnant women attending the East Boston neighborhood health clinic in USA [54]. Zielinska-Danch et al. (2007) reported a cutoff value of 550 ng/mL obtained from 327 participants in a Polish study [55]. The authors selected the cutoff value from a separation point in the bimodal distribution of urine cotinine in self-reported smokers and nonsmokers. Goniewicz et al. [38] reported 31.5 ng/mL as the cutoff value for distinguishing adult active from passive smokers based on ROC analysis of data from participants recruited for 6 different studies. Recently, Kim and Jung [56] obtained the optimum cutoff value (164 ng/mL) for urinary cotinine using the Korea National Health and Nutrition Examination Survey database (2008–2010, N = 11,629). The application of the urinary cotinine cutoff value provided sensitivity of 93.2% and specificity of 95.7%.

## 4. Discussion

### 4.1. Issues in Determining Cutoffs

As summarized in Table 1, saliva cotinine studies conducted to determine cotinine cutoffs varied widely with study population characteristics. For salivary cotinine, three studies [23,30,32] were conducted with participants in a smoking cessation clinical trial, while another study [27] was conducted with outpatients in a hospital in London. Also, as seen in Table 2, serum cotinine studies [35,36,42,43,46] were conducted on participants in a cardiovascular disease study, a clinical trial, or a health education program. Participants in smoking cessation clinical trials or health-related studies may possibly under-report their smoke exposure episodes. Past studies found that smokers tend to under-report their smoking episodes due to social norm pressures [57,58].

Three saliva studies [23,40,41] and two serum studies [45,47] were conducted on pregnant women. It is known that pregnancy status affects nicotine uptake and metabolism [59]. They found that the ratio of salivary cotinine per cigarette smoked during pregnancy (median, 3.53 ng/mL per cigarette) was much lower than the ratio after pregnancy (median, 9.87 ng/mL per cigarette). These findings suggest that the available cutoff value for adult pregnant populations is not applicable to the general population.

We noticed that three [30,31,41] of the eleven studies in Table 1 were conducted solely on white populations. Recently, Wells et al., (1998) reported that the misclassification rate of female occasional or regular smokers who were classified as never smokers was higher for U.S. minorities (15.3% or 2.8%) than for U.S. non-Hispanic whites (6.0% or 0.8%) [6]. The reported misclassification pattern for men was similar but slightly higher than that for females. Jarvis et al. (2008) also reported that the specificities of the given cutoff value were different between races: 98.4% in white participants and 90.2% in South Asian immigrants, indicating that a different cutoff value may apply for these Asian populations to achieve specificity comparable to that of the white participants. The consumption rate of oral tobacco products is very high in India, Pakistan, and Bangladesh. Furthermore, in some Asian countries, such as Japan, Korea, and China, women’s smoking, once tabooed, is dramatically increasing [60]. Using the NHANES data for 1999–2004 (3078 smokers and 13,078 nonsmokers), Benowitz et al. (2009) [37] reported serum cotinine cutoff values that differed with race/ethnicity: 5.92 ng/mL, 4.85 ng/mL, and 0.84 ng/mL for non-Hispanic blacks, non-Hispanic whites, and Mexican Americans, respectively. The estimated urinary cotinine cutoff values, 15 ng/mL and 60 ng/mL converted from the American serum cotinine cutoff value (3 ng/mL) and English saliva cotinine cutoff value (12 ng/mL), separately, were much lower than Korean urinary cotinine cutoff values. Thus, generalization of cutoff values based only on white populations may not be appropriate for other racial/ethnic groups. Furthermore, determination of country-, ethnic- and gender-specific cutoff values and validation of women’s smoking status are suggested.

From our review, we found a lack of consistency in study designs and reporting to facilitate comparison. For example, the gold standard used for sensitivity and specificity tests differed from study to study (i.e., self-reporting or cotinine test). In several studies, we found that the sensitivity dropped to lower than 80% from 90% or higher when the cotinine test, rather than self-reporting, was used as a gold standard with the same cutoff value selected [35,36,37,52] (Figure 1) indicating self-reporting may not accurately reflect their true smoking status.

With respect to culture, it is likely that many young female smokers reported themselves as smokers even though their smoking behavior was of low intensity which can also have the impact on the study determining a cutoff value.

Thus, we recommend providing sensitivity and specificity of the given cutoff value based on the two gold standards and the corresponding 2 × 2 tables, so that their estimation can be compared and validated. We also suggest reporting the sensitivity when specificity is 95%, or vice versa, for comparison of similar types of studies conducted in different times, populations, and regions. Indeed, such information from a large population-based study will be very valuable.

### 4.2. Drop in Cutoff Values over the Last 20 Years

Although it appears that serum, saliva or urine cotinine can be used to differentiate smokers from nonsmokers within each study, the generalization of any particular study is still limited because the sensitivity and specificity may vary with race, population characteristics, the tobacco products smoked and patterns of use, passive smoke exposure and the absorption path, suggesting that the optimal cutoff for the classification can also vary with population characteristics or regions and over time. This could be seen by comparing the 2 saliva cotinine cutoff values reported in 1987 and 2008 by Jarvis et al. in the UK (Table 1) [39] and the cutoff values obtained from NHANES 1988–1994 and NHANES data for 1999–2004 [37].

Jarvis et al. [39]’s recent cutoff value, 12 ng/mL, demonstrates a reduction of the salivary cotinine cutoff value in the UK population; from 14.2 ng/mL to 12 ng/mL over a period of 20 years. However, the difference was relatively smaller than the one observed in U.S. population seen below.

Benowitz et al.’s (2009) recent cutoff values for adults (3.08 ng/mL) and adolescents (2.99 ng/mL) based on ROC analysis of the NHANES data for 1999–2004 (9901 for adults and 5138 for adolescents) [37] were much lower than the earlier ones reported by Caraballo et al. (2001, 2004) using NHANES 1988–1994: 15 ng/mL for adults (N = 15,357) and 11.4 ng/mL for adolescents (N = 2107) [48,51]. Caraballo et al. also used ROC tests. These results indicate that the serum cotinine cutoff value in the U.S. population dropped over a period of 20 years. A reduction in the amount of tobacco smoking among smokers and passive smoke exposure among nonsmokers with increased the U.S. tobacco control policy efforts, including tobacco excise taxes, smoke-free workplaces, youth access laws, and increased tobacco control funding [61] over the past two decades may account for this drop in cutoff value.

Using a conversion factor (1.16) from serum to saliva cotinine, Jarvis et al.’s recent cutoff value for saliva obtained from the data of the HSE was compared with Benowitz et al. [37]’s recent serum cotinine cutoff value. Jarvis et al. [39]’s result was somewhat higher than the value obtained by Benowitz et al. [37] from the U.S. NHANES data sets. The difference in cutoff values between the two countries may be due to the difference in smoking prevalence in the two countries. According to the World Health Organization (WHO) report on Global Tobacco Epidemic [60], age-standardized adult smoking prevalence for the UK was 28.4% while that for the U.S. was 18.7%; The distribution of the tobacco smoke biomarker in nonsmokers would reflect the pattern of never smoking or passive smoking, while in active smokers it would reflect tobacco use. The distribution may change over time; with effective tobacco control, the distribution of values in nonsmokers would shift to the left and more nonsmokers would have a non-detectable level. With such a shift to the left, the cutoff point might also be shifted to the left, i.e., lower value.

Our review has several limitations. We mainly used the PUBMED database to extract articles. Other useful articles may exist in other databases. However, by searching the bibliographies of the articles included, we could increase the number of articles for our review. When we calculated sensitivity and specificity with self-reporting or cotinine tests using the provided 2 × 2 table, we assumed 100% accuracy for cotinine tests used in each study. We did not provide a cutoff value distinguishing passive smokers from nonsmokers. With evidence of harmful effects of third-hand smoke as well as second-hand smoke [62], a future review on cutoff value for passive smoke may be needed.

## 5. Conclusions

In this study, we examined studies that reported cotinine cutoff values and the potential challenges of applying a reported salivary or serum cotinine cutoff value to the general population. Providing country-specific cutoff values or multiple cutoff values of biomarkers for different exposure categories in large population groups (i.e., active smokers vs. passive smokers, passive smokers vs. nonsmokers) in a country may be a more desirable approach that would enable other researchers to compare and use the most relevant information for their purposes.

## Figures and Tables

**Table 1:** Salivary cotinine cutoff values, and methods of cutoff determination.

Author	Year	Ref.	Study Population Characteristics				Cutoff Value (ng/mL)	Number of Self-Reported		Method for Cutoff Determination
			Description	N	Age	Female (%)		Smokers	Non-Smokers	
McNeill et al.	1986	[29]	Students attending a girls’ comprehensive school in London, UK	508	11–16 years	100	14.7	173	335	2 × 2 table
Jarvis et al.	1987	[27]	Outpatients at St. Mary’s Hospital, London, UK	211	Mean age: 55 years	24.6	14.2	111	100	2 × 2 table: The cutoff value providing the highest numbers of correctly classified smokers and nonsmokers
Pierce et al.	1987	[31]	Residents randomly selected in a community, Australia	975	14 years or older	49.2	44	353	622	2 × 2 table
Stookey et al.	1987	[32]	Participants in a clinical trial for evaluating measures to facilitate smoking cessation, USA	236	NA	NA	10	216	20	Cutoff value was adopted from the study results of Benowitz (1983) [7]: “there was no nonsmoker with blood cotinine values greater than 10 ng/mL” (page 21)
Luepker et al.	1989	[28]	High school students randomly selected for survey in Minneapolis, MN, USA	263	17–21 years	NA	20	87	176	2 × 2 table
Etzel *	1990	[12]	Participants in 22 studies published between 1973 and 1989	NA	NA	NA	10	NA	NA	Cutoff value was selected after comparisons of the distributions of salivary cotinine concentrations with respect to smoking status between 22 study papers published between 1973 and 1989
Murray et al.	1993	[30]	Participants under routine care in a clinical trial of “Lung Health Study” in USA and Canada with evidence of early stage chronic obstructive lung disease	1498	35–60 years (mean: 48.5 years)	36	20	1345	153	Cutoff value was selected as the salivary cotinine level that provided the highest percentage of correctly classified smoking status
Boyd et al.	1998	[23]	Pregnant women in the Birmingham Trial II conducted at four public health maternity clinics in Birmingham, AL, USA	548	Mean: 24.6 years	100	24	441	107	ROC (receiver operating characteristics curve): The cutoff value providing the greatest percentage of correctly classified smoking status
Jarvis et al.	2008	[39]	Participants in the Health Survey for England: 1996–2004	24,332	All adults	NA	12	8808	15,524	ROC
Stragierowicz et al.	2013	[40]	Pregnant women in Polish Mother and Child Cohort Study	69	Mean: 26.4 years	100	12.9	19	50	ROC
Smith et al.	2014	[41]	Pregnant women in Southcentral Foundation’s Anchorage Primary Health Care Center: 2006–2010	376	Mean: 26.9 years	100	1.07	116	260	ROC

* Etzel did not provide gold standard information, but the cutoff value was included in this paper because the value was obtained after reviewing a large number of papers. ROC: receiver operating characteristics. NA: Not Available.

**Table 2:** Blood cotinine cutoff values and methods of determination.

Author	Year	Ref.	Study Population Characteristics				Cutoff Value (ng/mL)	Number of Self-Reported		Method for Cutoff Determination
			Description	N	Age	Female (%)		Smokers	Non-Smokers	
Williams et al.	1979	[42]	High school students participating in a school health education program. Blinded for purpose of blood collection in USA	118	14–17 years	53	3.0	21	97	2 × 2 table
Benowitz	1983	[7]	Participants in a test evaluating an elimination half-life for cotinine. They stopped smoking in a research ward (Average, 19.1 h; range, 10.9 to 37.0 h)	16	NA	NA	10	NA	NA	Authors selected the cutoff value from a range of concentrations among nonsmokers, and reported that “no nonsmoker had blood cotinine values higher than 10 ng/mL”
Slattery et al.	1989	[35]	Participants in (1) a cross-sectional study on dietary intake and hormone;	(1): 112	17 years or older	(1): male only	15	(1): 3	(1): 109	Authors selected the cutoff value by calculating 6% of the mean serum cotinine levels in smokers in the study
			(2) a case control study of squamous cell cervical cancer in Utah, USA	(2): 547		(2): female only		(2): 163	(2): 379	
Wagenknecht et al.	1992	[43]	Young adults in a cohort of cardiovascular disease study in USA	4984	17–30 years	NA	14	1540	3444	ROC
Pirkle et al. *	1996	[44]	Participants in the third National Health and Nutrition Examination Survey (NHANES), USA	10,270	4 years or older	50	15	NA	NA	Authors selected the cutoff value from a separation point in the bimodal distribution of serum cotinine in tobacco users and nonusers
Nafstad et al. *	1996	[45]	Pregnant women in the Oslo Birth Cohort, Norway	202	Mean: 30 years (Range: 19–43)	100	14	42 regular + 24 occasional	136	The traditionally used cutoff value (14 ng/mL) was chosen at the authors’ discretion
Heller et al.	1998	[46]	Followers among participants in the WHO MONICA (Monitoring trends and determinants in cardiovascular disease) project in 1987–1988, Germany	3661	TBA	50.9	15	1227	2434	Cutoff value was adopted from the study results of Wagenknecht et al., 1992 [43]
Pichini et al.	2000	[47]	Pregnant women attending the Hospital del Mar in Barcelona, Spain	404	TBA	100	14	136	268	Cutoff value was adopted from the study results of Nafstad et al., 1996 [42]
Caraballo et al.	2001	[48]	Adults in the third National Health and Nutrition Examination Survey (NHANES) 1988–1994, USA	15,357	17 years or older	53.8	15	4274	11,083	Cutoff value was adopted from the study results of Pirkle et al., 1996 [44]
Assaf et al.	2002	[49]	Adults those who conducted cotinine tests in Pawtucket Heart Health Program 1985–1986, USA	784	18–65 years	57.5	58 for male (M)	131 (M)	172 (M)	ROC
							30 for female (F)	141 (F)	279 (F)	
Seccareccia et al.	2003	[50]	Providers of serum samples among participants in the project of MATISS (Malattie cardiovascular Aterosclerotiche, Istituto Superiore di Sanità), Italy	3379	20–79 years	39.5	15	977	2402	ROC
Caraballo et al.	2004	[51]	Adolescents in the third National Health and Nutrition Examination Survey (NHANES) 1988–1994, USA	2107	12–17 years	53.8	11.4	213	1894	ROC
Martinez et al.	2004	[36]	Participants in a dietary trial on adenoma recurrence, Phoenix, AZ, USA	824	40–80 years	31	20	95	729	2 × 2 table
Benowitz et al.	2009	[37]	Participants in the National Health and Nutrition Examination Survey (NHANES) for 1999–2004, USA	9901 for adults,	20 years or older	50.6 for adults	3.08 for adults	2340	7561	ROC
				5138 for adolescents	12–19 years	49.6 for adolescents	2.99 for adolescents	515	4623	
Jeemon et al.	2010	[52]	Participants in the cardiovascular disease surveillance program at New Delhi, India	426	18 years or older	TBA	40.35	142	284	ROC

* Benowitz (1983) [7], Pirkle et al. (1996) [44], Nafstad et al. (1996) [45], did not provide sensitivity and specificity values, but the cutoff value was included in this review because the values were obtained after reviewing a large number of study populations or the cutoff value was referred by other studies.

**Table 3:** Urine cotinine cutoff values and methods of determination.

Author	Year	Ref.	Study Population Characteristics				Cutoff Value (ng/mL)	Number of Self-Reported		Method for Cutoff Determination
			Description	N	Age	Female (%)		Smokers	Non-Smokers	
Hoffmann et al. *	1984	[34]	Volunteers joined a study on uptake of sidestream smoke	NA	NA	NA	55	NA	NA	The value was obtained from urine samples that were collected when saliva nicotine levels returned to baseline levels (i.e., 5 h after study subjects were exposed to passive smoke in closed chamber (280 mg/m^3^ for air nicotine concentration) for 1 h)
Riboli et al. *	1990	[53]	Married nonsmoking women from 10 countries	1369	Age: 42–60 years	NA	50	NA	NA	Cutoff value was chosen as the value that provided 3.4% misclassification. It was also compared with the study results of Hoffmann et al. (1984) [34]
Pickett et al.	2005	[54]	Pregnant women attending the East Boston neighborhood health clinic, USA between 1986 and 1992 with allowance of multiple visits	998	19 years or more	NA	200	1272	3566	ROC
Zielinska-Danch	2007	[55]	Volunteers living in Sosnowiec, Poland	327	19–60	57.2	550	111	216	Authors selected the cutoff value from a separation point in the bimodal distribution of urine cotinine in self-reported smokers and nonsmokers
Goniewicz et al.	2011	[38]	Smokers from three different studies conducted in; San Francisco, CA, USA, Silesia, Poland, and Pittsburgh, PA, USA Nonsmokers from the other three studies conducted in USA, Poland, and Mexico	601	18 years or older	52.7	31.5	373	228: passive smokers only	ROC
Stragierowicz et al.	2013	[40]	Pregnant women in Polish Mother and Child Cohort Study	69	Mean: 26.4 years	100	53.0	17	52	ROC
Kim and Jung	2013	[56]	Participants in Korea National Health and Nutrition Examination Survey (KNHANES) for 2008–2010, Korea	11,629	19 years or older	55.5	164	2547	9082	ROC

* Hoffmann et al. (1984) [34] and Riboli et al. (1990) [53] did not provide sensitivity and specificity values, but the cutoff values were included in this review because the values were one of few available values referred by other studies or obtained after reviewing a large number of study populations, respectively.
